# Social Workers in Animal Shelters: A Strategy Toward Reducing Occupational Stress Among Animal Shelter Workers

**DOI:** 10.3389/fvets.2021.734396

**Published:** 2021-11-10

**Authors:** Janet Hoy-Gerlach, Mamta Ojha, Phil Arkow

**Affiliations:** ^1^School of Social Justice, University of Toledo, Toledo, OH, United States; ^2^National Link Coalition, Etowah, NC, United States

**Keywords:** animal shelter staff, occupational stress, veterinary social work, compassion fatigue, human animal support services

## Abstract

Animal shelter workers (ASWs) are at disproportionate risk of moral injury, secondary trauma, compassion fatigue, and burn-out. While there is an emerging body of literature developing on understanding the nuances of these experiences for ASWs, little work has been done on developing strategies to ameliorate occupational stressors and the negative effects of such for ASWs. Within this paper, occupational risks and protective factors for ASWs are summarized, and the emergence of social work within animal shelter settings as one strategy for helping to ameliorate the occupational stress experienced by ASWs is delineated.

## Introduction

Estimates suggest that ~6.3 millions companion animals enter animal shelters every year in the U.S. and about 920,000 of them are euthanized (1). While there are tremendous rewards involved in working with shelter animals, individuals who do such work are also exposed to animal suffering and death and are at disproportionate risk of negative mental health outcomes. Individuals engaged in animal shelter-related work are at a disproportionately high risk of secondary trauma (2), and have a five times greater risk of developing Post-Traumatic Stress Disorder as compared to the national average (3). Individuals who worked as animal control workers were in the highest risk occupational risk group—along with police officers and other protective workforce roles (excluding armed forces/military) for workplace suicide (4).

Given the disproportionately high mental health risks faced by animal shelter workers—henceforth referred to as ASWs—there is a dire need to develop multiple strategies that mitigate these risks and mobilize protective factors for ASWs. ASWs are not the only occupation involving work with animals that are at disproportionate risk of experiencing such negative occupational effects. Similar to ASWs, veterinarians experience compassion fatigue (2, 5, 6), moral stress, moral distress or moral injury (7–9), secondary traumatic stress (10, 11), and burnout (2, 7), and are at a disproportionately high risk of suicide compared to the general population (12, 13). Specifically, veterinarians were found to be two to three times more likely to complete suicide as compared to the national average (12). Due to such occupational risks experienced by veterinarians, social workers have been increasingly incorporated into veterinary practice, and Veterinary Social Work has emerged as a unique area of social work practice (14).

Having a mental health provider or a social worker on staff for consultations, sessions, or de-briefs could similarly support the needs of ASWs (15). The risk factors and negative outcomes faced by ASWs are multi-faceted and necessitate a range of responses; a small number of animal welfare organizations in the United States and Canada have begun to incorporate social workers on staff or as interns in an attempt to ameliorate these adverse situations. For example, the Arizona Humane Society added a social worker to staff as a “resource navigator” to elevate its intervention efforts to assist pet owners in crisis with sustainable, long-term solutions that would prevent them from unnecessarily surrendering their animals. “We learned early on that we can't care for pets if we don't care for those on the other end of the leash as well… the people,” said Dr. Steven Hansen, President and CEO. The Resource Navigator also works closely with admissions and field rescue teams to provide crisis support and training to staff assisting on the front lines during crisis situations such as homelessness, financial hardships and domestic violence (16).

Social work offers one potential resource for ASW entities in helping to address both individual- and organization-level risk factors for occupational stress and related negative outcomes. The purpose of this article is two-fold: to explore how incorporation of social work is emerging as one potential strategy within animal welfare organizations to help mitigate the occupational stress experienced by ASW staff; and to offer specific approaches for expanding the presence of social workers in ASW settings.

## Overview of Occupational Stressors and Protective Factors in Animal Shelter Work (ASW)

### Occupational Stressors in ASW

The occupational stressors experienced by ASWs put them at disproportionately high risk for a group of interrelated negative outcomes: moral injury; secondary trauma stress; burnout; compassion fatigue; lower job satisfaction; and job turn-over (3, 5, 17, 18). There is speculation that the core factor among these is what Andrukonis et al. (19) referred to as “moral distress,” defined as that which “…occurs when a person engages in, bears witness to, fails to prevent, or learns about acts that transgress the individual's moral code…” (p. 2). The ASW—often drawn to this occupation due to caring about animals—may find it difficult to justify killing and suffering of shelter animals on moral grounds, resulting in experiences of psychological distress identified in the literature as *moral distress* or *moral injury* (8, 20). ASWs who directly participated in euthanizing animals were at significantly higher moral injury rates, suggesting that proximity to the incidence and decisions that rest solely on employees are likely to increase the incidence of negative impact on workers (3).

In addition to moral distress/injury, ASWs are disproportionately exposed to primary and secondary trauma. Performing/witnessing euthanasia can be a source of primary trauma for ASWs. Most ASWs enter shelter work because they care for animals, and their engagement in and exposure to euthanasia of healthy animals has been identified as a “caring-killing paradox” (17, 21, 22) that can induce perpetration-induced traumatic stress in these workers, and they are at a higher risk for developing Post-Traumatic Stress Disorder (23). In their seminal work, Reeve et al. (17) conducted an exploratory study to examine euthanasia-related strain among ASWs. Data was collected in two waves and results suggested that direct ASW involvement in shelter animal euthanasia caused significant stress and had a significant impact on their well-being and ability to function. This job-related stress among ASWs in the study was also associated with increased work-to-family conflict and somatic complains as well as lower levels of job satisfaction (17).

ASWs are also frequently exposed to animals who have been neglected, injured and abused; this comprises a form of secondary traumatic stress. Figley (24) defined secondary traumatic stress as the “natural and consequential behaviors and emotions resulting from knowing about a traumatizing event experienced by a significant other [animal] and the stress resulting from helping or wanting to help a traumatized or suffering person [or animal]” (p. 7). Simply put, secondary traumatic stress is a reaction an ASW can have upon being exposed to an animal's trauma; the Diagnostic and Statistical Manual of Mental Disorders, 5th Edition (DSM 5) recognizes exposure to secondary trauma as a valid stressor criterion in diagnosing Post-Traumatic Stress Disorder (25).

In addition to negative outcomes related to animal harm exposure, burnout is related to chronic organizational/work life stressors that result in exhaustion, cynicism, and inefficacy (19). Leiter and Maslach (26) identified six areas of work life that are associated with burnout that have informed much of the subsequent research in this area: workload, control, reward, community, fairness, and values. Poor working conditions in many animal shelter environments put high physical and psychological job demands on ASWs (15), resulting in a higher incidence of stress among them (3). Conflicts with supervisors and low pay have also been identified as organizational stressors for ASWs (27).

While burnout is related to feeling apathetic and hopeless due to chronic organizational stressors, compassion fatigue pertains to the physical, emotional, and spiritual exhaustion from taking in the difficulties and suffering of others (28). Compassion fatigue encompasses both burnout and trauma and is a phenomenon frequently present in those in occupations that involve caring for others (19, 28). Andrukonis et al. (19) define compassion fatigue as “…a state of emotional dysregulation, comprising secondary traumatic stress and burnout, that negatively influences individuals in caring professions…” (p. 2). Euthanasia-related stress and degree of exposure to cruelty and neglect cases have been identified as the strongest risk factors for compassion fatigue among ASWs (2).

### Protective Factors and Strategies to Reduce Risk in ASW

Research on protective factors against occupational stressors in ASW has been limited. Increased live release rates (LRR)—identified as the percent of animals that leave shelters with a positive outcome—were positively associated with lower moral injury among ASWs (3). LRR were also associated with compassion satisfaction, a concept linked inversely to compassion fatigue and relating to feeling satisfied, competent, and supported by peers and management in the work of caring for others (28). However, LRR were also associated with secondary trauma, moral injury, and burnout among ASWs, suggesting that while more positive outcomes in shelters are associated with increased job satisfaction, there may also be associated stress involved in obtaining such (3).

An intrinsic motivation to help animals, affinity with animals in general, and attachment to shelter animals have been identified as resiliency factors against compassion fatigue among ASWs (29). ASWs who identified their work as meaningful and felt that it helped them to make a difference in these animals' lives appeared to have protective effects from the negative impacts of stressful situations at work (29). Study participants also reported social support from animal shelter workers as a resiliency factor and recognized the need for ongoing self-care (29).

Social support from other ASWs as an important resource in mitigating stress for ASWs was initially suggested by Baran et al. (27) who identified seeking conversations, interactions and relationships with “insiders”/other employees within one's occupation group as an important supportive resource in coping with occupational stressors, particularly when one's occupation entailed “dirty” tasks such as euthanasia that are stigmatized by the public. Anecdotally, the lead author of this publication observed the importance of “insider support” when arranging a self-care and stress reduction training for ASWs by their employee assistance provider. Numerous ASWs reported in their evaluations of the training that they felt the employee assistance provider presenter could not understand their stressors because she hadn't experienced things like the volume of unwanted cats occurring annually during the Spring “kitten season” and performing euthanasias. The following year, a similar training was offered at the request of ASWs, but the presenter for the second training was a Veterinary Social Worker from a busy veterinary teaching hospital who had been present for many animal trauma cases and euthanasias. While the basic content of the two trainings was very similar, the ability of the trainers to connect with the ASWs was dramatically different, as evidenced by comments in the training evaluations. Multiple evaluations from the second training had comments indicating that the attendees felt much more understood and effectively supported by the Veterinary Social Work presenter.

Rank et al. (30) identified a reluctance among at-risk individuals in animal-caring professions, including animal control and animal shelter employees, to seek interventional support for compassion fatigue. These employees are often unable to articulate their needs or give narrative to their experiences which include traumatic histories of their patients, bleak working conditions, high demands, minimal resources to cope with an endless stream of homeless animals, conflicts in the workplace and at home, and a lack of empathy from the public. They described a three-phase “training-as-treatment” protocol that was found to be both statistically and clinically effective in helping professionals lessen symptoms of compassion fatigue and enhance their resilience. Results of a study of 57 professionals found the protocol had a sustained ameliorative effect upon respondents' compassion satisfaction, burnout, trauma recovery, and state and trait anxiety. The intervention was said to offer “an ounce of prevention and a pound of cure to the symptom-saturated population of non-human-animal care professionals” (p. 55) and deemed to be worthy of further study.

In their “training-as-treatment” study, Rank et al. (30) described numerous implications for social work practice; training-as-treatment was conceptualized as a therapeutic intervention that social workers can utilize to reach more professionals such as ASWs who are at risk for compassion fatigue and who may be reticent to present for treatment otherwise. Rank et al. (30) concluded that social workers can play a major role in helping ASWs cope with compassion fatigue related to their stressors. Examples of such stressors include but are not limited to: witnessing animal suffering, awareness of euthanasia, having a responsibility for life, working with distressed and abusive clients, receiving negative public perceptions particularly *vis-à-vis* open-admission shelters, and having intense attachments to animals under their care.

Given the parallels between the occupational stressors experienced in veterinary settings and those experienced in ASW, and the positive impact Veterinary Social Workers have reportedly had in helping to ameliorate occupational stressors in veterinary practices (14), it is worth exploring how to expand the Veterinary Social Work model to include animal care and control professionals.

### Trends in Veterinary Social Work That Are Applicable to ASW

The recognition of needs and opportunities for applying a social work model to assist professionals who work with animals was initiated with the notion of Veterinary Social Work, which entails training social workers to attend to human needs in veterinary clinics and hospitals (31). Such work generally revolves around four primary activities: grief and bereavement counseling for clients whose pets have died or are facing euthanasia; therapeutic animal-assisted interventions; addressing links between abuse of animals and potential co-occurring child maltreatment, domestic violence or elder abuse; and compassion fatigue and conflict management for staff (31). Veterinary Social Workers work on the individual one-on-one level, or on a larger scale working with communities or influencing public policy, and facilitate problem-solving, decision-making and psychoeducation about issues that involve animals to improve circumstances for the people involved, thereby benefiting the animals as well (32). All of these have potential applications as well in the animal shelter environment (33, 34).

Through having a knowledge base and intervention skill set in the facilitation of planned wellness-focused change at both the individual- and organization/social environment-levels, social workers have increasingly collaborated with veterinary professionals to help alleviate occupational stressors and risk factors over the past two decades (14). Veterinary Social Work has emerged as a specialized area of social work practice that specifically addresses the human issues that emerge within the human-animal relationship.

In 2002, under the leadership of social worker Dr. Elizabeth Strand, The University of Tennessee College of Veterinary Medicine and College of Social Work collaboratively established the first Veterinary Social Work certificate program within the graduate social work program—as a credential for practicing social workers—within the United States. (14). The Veterinary Social Work certificate program is completed by social workers who have MSWs and are currently working within the social work field.

The recognition of a need for Veterinary Social Workers within veterinary settings has continued to expand since the formalization of Veterinary Social Work Education (14). While there is no official count of the number of practicing Veterinary Social Workers, the University of Tennessee's Veterinary Social Work program has a mailing list of about 1,000 individuals (34, 35). Many Veterinary Social Workers have worked in private clinics and particularly in veterinary teaching hospitals, including Colorado State University, the University of Georgia, Michigan State University, the University of Minnesota, North Carolina State University, the Ohio State University, the University of Pennsylvania, Purdue University, the University of Tennessee, Tufts University, the Virginia-Maryland College of Veterinary Medicine and the University of Saskatchewan. Their role generally is two-fold: to address compassion fatigue among veterinary staff, and to address emergent psychosocial issues with human clients who are bringing their animals for treatment (36) , by providing expertise in these areas they enable staff to focus on their expertise in practicing veterinary medicine.

Private emergency and specialty veterinary practices are similarly increasingly hiring Veterinary Social Workers to address such issues (14). For example, the Veterinary Social Work team at BluePearl Specialty and Emergency Pet Hospitals, a veterinary provider organization with over 100 veterinary hospitals across the United States, developed a Wellness Ambassadors Program as a strategy to help mitigate occupational stressors and mental health risks among veterinary staff, create trained wellness champions across the organization, and promote a culture of health and well-being (37). Wellness Ambassadors will be trained in Mental Health First Aid, an evidence-based public education program similar to physical first aid in how it is conceptualized. Peer “insider support” from other veterinary staff members is a crucial component.

There is now international recognition of Veterinary Social Work being cast as not just limited to veterinary practices, but rather as an area of social work practice that supports and strengthens interdisciplinary partnerships that attend to the intersection of humans and animals (38). The International Association of Veterinary Social Work was formed in 2020 and serves as the professional organization for social workers across the globe who are engaged in this broad definition of veterinary social work, which is inclusive of social workers who address human-animal interaction issues across a wide range of practice settings and contexts (38). The organization's mission is to support and promote professionals who tend to the human needs that arise in the relationship between humans and animals by creating and maintaining professional standards, encouraging research, and advocating for a better world for all species (38). This broad conceptualization of Veterinary Social Work is inclusive of social work practice with humane societies and animal shelter settings.

### Historical and Current Applications of Social Work in ASW

The addition of social workers to animal shelter environments represents a return to the historical connections linking social work-based child protection and animal protection (34); the first child abuse cases in the U.S. were handled by humane organizations and led to the founding of the first society for the prevention of cruelty to children, connecting an encompassing concern for equity and social justice in an attempt to use new Darwinian thought to lessen the distance between humans and animals (39). From the 1870s onward, child welfare and animal welfare work often overlapped: pioneering social reformer Jacob Riis (40) described the American Humane Association (AHA), organized in 1877 as a federation of local organizations, as protecting “the odd link that bound the dumb brute with the helpless child in a common bond of humane sympathy” (p. 150). Child protection closely aligned the fledgling animal protection movement with other social reform and social justice movements concerned with cruelty, violence and the social order (41, 42). For example, the Illinois SPCA (Society for the Prevention of Cruelty to Animals), founded in 1869, changed its name in 1877 to the Illinois Humane Society to more accurately represent an expanded focus that had come to include the prevention of cruelty to children (43). By 1922, 307 of the AHA's 539 animal protection organizational members also protected abused children as part of the same humanitarian continuum of care (44). Until the Great Depression, more humane societies operated orphanages than ran animal shelters, usually as a secular alternative to the work long done by religious organizations and the infamous public workhouses described by Dickens. The Connecticut Humane Society, the last humane society to handle child protective services in lieu of a government agency, did not relinquish that role until 1966 (45).

An emerging movement in animal sheltering work referred to as Human Animal Support Services (HASS) is revisiting the interconnectedness of human and animal welfare service provision. A coalition of 37 pilot shelters in the U.S. and Canada is implementing a community collaborative model that entails partnering with local human service providers to keep animals in their homes and communities, rather than in shelters (46). This aim is accomplished through empowering pet owners to find solutions for common human-animal challenges, uncovering the root causes of animal problems while conducting field services, and building community partnerships that treat people and animals as a family unit (46). HASS shelters offer case management (a social work function) that provides resources and support for struggling pet owners, such as pet food banks, affordable veterinary care, mental health care, temporary foster care for pets when owners are in a health or economic crisis, and housing help (46). These likewise are all within the sphere of traditional social work activities.

Many animal shelter jobs, though not specifically designated as a social work position, lend themselves to individuals with social work training and perspective. These include positions in human resources, adoption counseling, grief counseling, client support services, community assistance services, foster care placement, volunteer coordination, and staff training, as well as senior management positions in executive leadership and development. Many shelters offer foster care for pets displaced by owners who are homeless, victims of disasters or survivors of domestic violence who need counseling and support. Numerous larger animal shelters have veterinary departments specializing in animal shelter medicine for their own animals, and low-cost services for the public, where social workers can easily assume the same responsibilities and functions as Veterinary Social Workers who work within in private practices and university teaching hospitals.

A handful of animal care and control shelters across the United States and Canada have begun to formally incorporate social work roles, to support people coming to shelters for assistance and, to a lesser extent, to address the occupational stress related to ASW. In addition to the previously-mentioned “resource navigator” at the Arizona Humane Society, other social work and community caseworker positions and internships have reportedly opened up at organizations (47) including: Animal Haven in New York City; the Animal Humane Society in Minneapolis; the Animal Rescue League of Iowa; the Denver, Colo. Animal Shelter; the Denver, Colo. Dumb Friends League; the Houston SPCA; the KC Pet Project in Missouri; the Lawrence, Kans. Humane Society; the Lifeline Animal Project in Georgia; the Oregon Humane Society; the Pima County, Ariz. Animal Care Center; the Royce-Hurst Humane Society in Colorado; and the Santa Cruz County, Calif. Animal Shelter, among others. A few similar positions and internships have been reported anecdotally in Canada and Australia. The Toledo Humane Society in Ohio has had graduate level social work interns since 2010, with one of the primary internship tasks being to address staff wellness and amelioration of occupational stressors (34).

### Opportunities to Integrate Social Work Into ASW

Despite a history of interconnection, non-profit animal welfare and governmental animal control agencies have often operated in isolation outside the purview of human services agencies (48). Consequently, interagency cooperation, and cross-training have been minimal, and cross-reporting of animal abuse by child protection workers and of child maltreatment by humane agents and animal control officers, though required or permitted by several states' laws, has been sporadic (49).

The isolation of animal care and control from other social service agencies creates situations where ASWs are often unaware that their counterparts in human services experience similar stressors and that there are social work and mental health resources available to assist them. This lack of knowledge and coordination among community systems constricts the potential for creative and effective collaborations and can increase the risk of harm to people and animals in situations where both human and animal abuse co-occur.

Formally integrating social work into animal care and control shelters would not only continue the historical precedent and solidify the emerging collaborative relationships forming through HASS endeavors, but would also create a new linkage between humane and human services that could reduce a silo effect in which cross-disciplinary and trans-species community collaborations rarely occur.

Social workers can facilitate bridging these segregated service delivery systems through the profession's longstanding commitment to community-level action, intervention and change. Social workers can work through animal shelters to organize species-spanning community coalitions, link organizational champions, and connect consumers and professionals for the well-being of underserved and at-risk individuals and family members (50).

Animal control and humane officers frequently have access to pet owners' homes in the course of their investigations, and in the process may observe conditions detrimental to the welfare of children, youth and others. In addition, cruelty investigations which result in the removal of animals from a home could be an additional stressor on the family system and could lead to increased risk for vulnerable family members. Social workers can train shelter personnel on the intersectionality of animal abuse and human violence and the procedures for making referrals to social services agencies (33). They could also play crucial roles in the investigation of animal hoarding cases and the creation of multidisciplinary animal hoarding task forces (51).

Some animal shelters, often working with juvenile and adult detention centers, have implemented animal-assisted therapy interventions where individuals who have offended, or who are at risk, train dogs with behavior problems who are at risk of being euthanized. Using positive reinforcement techniques, these programs teach teamwork, non-violent conflict resolution and collaboration skills to save animals' lives and modify the behaviors of abusive and traumatized individuals (52).

Other untapped social work opportunities in animal shelters might include:

- strengthening collaborations with domestic violence shelters and mobile meals programs;
- directing and Expanding pet visitation programs into long-term care facilities and animal-assisted interventions for at-risk populations;
- developing pet loss grief support groups;
- developing safety net supportive programming for individuals who experience a medical, economic or housing crisis that temporarily makes it difficult to keep an animal;
- defusing contentious confrontations with shelter clients by resolving customers' complaints and needs for services; and
- connecting pet owners with community resources, such as low-cost pet and veterinary services, animal behavioral counselors, pet food banks, and social services agencies (33).

Social work support offers promise as a resource in animal shelter settings. Animal shelters appear poised for such systemic change. The service philosophy in the animal shelter community is evolving to recognize that treating the symptoms of animal welfare problems, such as animal homelessness, abuse and neglect, is only a stopgap solution: to be truly effective, underlying community and family stressors must be addressed (53). Identification and intervention with community and family stressors lies squarely within the scope of practice of social work, and social workers can thus readily help to address such issues in ASW.

### Building a Framework to Incorporate Social Workers Into ASW

In order to implement these concepts and incorporate social workers into the animal shelter environment, several key developments must occur to make what is currently a novel and unevaluated concept into a more widely recognized and supported area of social work practice. These include but are not limited to:

- Developing and expanding human-animal bond content in social work education programs;
- Establishing Veterinary Social Work as a specialty area of social work practice;
- Encouraging social work field placements and internships at animal shelters;
- Encouraging the development of social work internships in animal shelter environments;
- Expansion of human-animal interaction content in social work continuing education; and
- The promotion of social work positions in animal shelter settings by national animal care and control organizations.

#### Development of Human-Animal Interaction-Related Curriculum Content in Social Work Degree Programs

A serious gap exists in the professional training of social workers with regards to recognizing the bonds and challenges that emerge in the relationships people have with animals (51). According to the American Pet Products Association (54) annual demographic survey—the largest demographic survey of those with companion animals in the United States−70% of U.S. households report having at least one companion animal. The majority of clients encountered by social workers in the United States are thus likely to have one or more companion animals. As of this writing, the National Link Coalition (55) has identified only 27 schools of social work, out of 889 accredited BSW and MSW programs in the U.S., where human-animal interactions are formally addressed in the curriculum. While social workers are trained to honor diverse client definitions of family, more professional training is needed to acquaint traditionally humancentric social workers in working with multi-species families and the resources that support them. The Council on Social Work Education, as the national accreditation body for social work programs in the United States, could develop and provide resources to support the development of human-animal interaction content for infusion into schools' curriculum.

Expanding awareness of the impact of animals and animal-related work in people's lives across social work settings can begin with something as simple as routinely including questions about pets in intake forms, assessments, and definitions of family support systems (51, 56, 57). As assessing clients' needs is an important step in developing the best plan to solve clients' problems, including pet protective factors in clients' ecologies should be considered a relevant environmental factor in social work practice theory (58). Collecting information about all the pets and humans in a family communicates interest and concern for the whole family and demonstrates an integrated approach to care in planning appropriate interventions and preventive care. By routinely considering human-animal relationships in interventions and assessments and working collaboratively with community resources—such human-animal support services at animal shelters and accessible veterinary services—that can help resolve clients' animal-related concerns, social workers can be more holistic and effective in resolving clients' needs and challenges and in preventing further abuse of vulnerable members of families and communities (33, 51). Such inclusion of human-animal interaction considerations within routine social work practice may also help to prevent surrenders of pets to animal shelters due to lack of resources by proactively linking people with the supports needed to keep their companion animals, ultimately helping to mitigate this aspect of ASW occupational stress.

#### Establishment of Veterinary Social Work as a Specialty Area of Social Work Practice

In addition to expanding human-animal interaction content as foundational knowledge in social work degree programs, Veterinary Social Work needs to be established as a specialty area of practice. The establishment of the International Veterinary Social Work Association in 2020 was an important first step in creating a specialty practice area focused on developing professional expertise related to addressing human needs that emerge in human-animal interaction.

Educational opportunities for post-graduate specialization in Veterinary Social Work also need to be expanded. Currently only one school—the afore-mentioned certificate program at the University of Tennessee—explicitly specializes in Veterinary Social Work. The University of Tennessee Veterinary Social Work program encompasses a broad conceptual view of Veterinary Social Work, primarily focusing on practice in veterinary settings but acknowledging a wide range of settings in which human needs intersect with animal health and welfare. The vision of VSW-CP [Veterinary Social Work-Certificate Program] is at the University of Tennessee is “to produce professional social workers knowledgeable in the practice and skills necessary to help people through human animal relationships in a variety of settings [emphasis added] and through a variety of micro and macro practice methods” (59). This program could be expanded to have more explicit content on emerging opportunities in animal shelters and could be replicated through partnerships with social work programs across the United States.

#### Encouraging Social Work Field Placements and Internships at Animal Shelters

As part of an expanded inclusion of human-animal relationships in general social work education and the establishment of Veterinary Social Work as a practice specialty, field placement directors in social work programs could recommend community animal shelters as fruitful venues for field placements and internships. Some schools of social work are already recognizing field placement opportunities for MSW candidates working with veterinary facilities, animal shelters, agencies encountering animal hoarding and other traumatic situations, and people working with animals in domestic violence facilities, schools and human healthcare agencies (60).

Since 2010, graduate social work students have completed internships for academic credit at the Toledo Humane Society in Ohio to help reduce staff occupational stress, and to help address the human issues emergent in the provision of animal welfare services (34). The interns, using the social work change facilitation skill set, built relationships with ASWs and developed and implemented numerous interventions aimed at ameliorating occupational stress and risk factors, including:

- Shadowing staff and participating in shelter departments to learn shelter operations and organizational culture and build relationships with ASWs;
- Assessing with shelter staff their stressors and strengths and what they felt would be most helpful in reducing stress;
- Based on staff input and feedback, planning, implementing and evaluating a “Wellness Week” at the shelter that entailed a lunch and daily stress-reducing group activities that included donated lunches, therapeutic massage, painting instructors, and creating spaces for staff to connect with each other and provide informal peer support;
- Working toward building a culture of wellness through crafting and sending weekly emails which incorporated ASWs' strengths, interests, and ideas;
- Supporting shelter staff as needed or requested with interactions with the public;
- Providing consultation and psychosocial support to staff as needed or requested;
- Providing information and referrals to address human needs that emerged in their interactions with the public; and
- Collaborating with social services entities to help train human service professionals on cross-reporting of animal cruelty, child abuse, and elder abuse (particularly relevant based on recent legislation in Ohio that mandates cross-reporting).

#### Expansion of Human-Animal Interaction Content in Social Work Continuing Education

Continuing education programming that encompasses human-animal support content for practicing social workers can be expanded and provided through entities that provide ongoing continuing education credits, such as the National Association of Social Workers (NASW) and its state chapters. There is already some momentum in this area; NASW state chapters in New York and Ohio have ongoing active working groups that offer continuing education workshops and other resources to support the practice of social work that is inclusive of human-animal interaction issues. Such trainings better equip social workers to provide quality care and support for humans who have companion animals in their social systems (30), as well as for those working in animal health or welfare who face disproportionate health risks due to occupational stressors.

#### Promotion of Social Work Positions in ASW Through National Animal Care and Control Organizations

Recognizing stressors (61) and high suicide rates among the veterinary profession, (62) the American Veterinary Medical Association has taken a leadership role in promoting self-care and well-being in the workplace. This campaign, which includes animal shelter veterinarians, could be adapted and modified to meet the needs of animal shelters. It could be promoted by the various national organizations which provide training and best-practice guidelines for animal shelters, such as the ASPCA, the Association for Animal Welfare Advancement, the Association of Shelter Veterinarians, Humane Society of the U.S., and the National Animal Care and Control Association.

Such an emphasis on wellness could easily include the addition of full- or part-time or contracted social work services. Given the chronic budget constraints faced by local animal shelters, such programs would conceivably require external funding sources from the philanthropic sector. Charitable organizations dedicated to the promotion of human and/or animal welfare could be made aware of the potential for animal shelter social work to save lives and help make humane societies' working environments truly humane.

### Additional Research Needs

There is admittedly an unfortunate paucity of research that targets occupational stressors inherent to animal welfare organizations (15). The limited literature that does exist regarding animal shelter workers is primarily focused on the assumption that euthanasia is the predominant source of occupational stress (17, 27). There is more literature on the occupational stressors impacting veterinarians, including challenging clients with unrealistic demands, low pay, long and irregular work schedules, insufficient staffing levels, voluminous caseloads, negative public perceptions, physical risks from aggressive animals, and frequent contact with death and dying (13, 62–65). It may be surmised that many of these same conditions can apply to ASWs and additional research is needed to examine these conditions in the animal shelter environment, their impact on employee satisfaction, and the potential for social work to alleviate resulting problems.

Moral injury has become an emerging topic in the animal care community. Future studies should evaluate potential moral stressors specific to animal care work. A better understanding of the causes of ASW distress will allow for better-informed intervention methods (3).

Staff inclusion in decision-making by management related to euthanasia has been identified by frontline staff as a way to decrease euthanasia-related occupational stress (66). However, in a later study, there was not a reduction in euthanasia-related stress among ASWs who identified having significant decision-making input related to euthanasia (3). These findings suggest a more nuanced investigation relating to decision-making input and support is likely necessary to determine how these may serve as protective factors for ASWs.

While most social workers in animal shelter settings have not as yet primarily focused on occupational stress reduction of ASWs, it is likely that preventing animal intakes by linking people with resources to keep their pets may have an ameliorating effect on ASW occupational stress; this is an area needing future research.

The effectiveness of the limited number of Veterinary and Animal Shelter Social Workers has never been adequately evaluated. Research is needed to evaluate the effectiveness of such positions using such metrics as employee retention, job satisfaction, client satisfaction with services, number of grief support and counseling sessions conducted, and other empirical indicators.

## Conclusion

If we care about the animals, we must also care about the workers who care for them and take steps to ameliorate ASW occupational stress (67). Given the multi-faceted nature of the high risks and level of stress endured in ASW, and the value of the work they do in safeguarding the well-being of animals in our society, it is crucial that a range of strategies are put into place to help ameliorate the risks to the well-being of ASWs. While knowledge is building on the nature of these occupational stressors and their negative outcomes, development of approaches for reducing them has been slower.

Incorporating social work practice within animal shelter settings has numerous applications for alleviating suffering and promoting well-being for humans and animals (51). Arkow (33) described Veterinary Social Work as the human side of veterinary medicine and the animal side of social work. Extrapolating from how social workers in veterinary settings are helping to ameliorate occupational stress for veterinary staff, the role of social workers in humane societies and animal shelters is a potential strategy that needs to be investigated and refined as a support for ASWs. Veterinary Social Workers could assist animal shelters whose staff are trained in animal welfare and behavior but are less familiar with the problems existing at “the other end of the leash.”

To maximize the potential of social work as a stress alleviating strategy in ASW, a framework for incorporating social work in ASW settings must be created. Specific components of such a framework include but are not limited to:

- Developing and expanding human-animal bond content in social work education programs;
- Establishing Veterinary Social Work as a specialty area of social work practice;
- Encouraging social work field placements and internships at animal shelters;
- Encouraging the development of social work internships in animal shelter environments;
- Expansion of human-animal interaction content in social work continuing education; and
- The promotion of social work positions in animal shelter settings by national animal care and control organizations.

Through direct supportive work with ASWs and ASW organizations, as well as systems-level coalition/policy work and reducing human client-related issues *via* provision of HASS services/referrals which may serve to indirectly reduce ASW stress, social workers offer promise as a stress-reducing presence in the lives of ASWs.

## Author Contributions

JH-G: developed concept for this paper and overall outline and writing of introduction, social work and veterinary social work sections, editing of literature review, and integration of all sections. PA: provided substantive revisions with broadening of article scope, strengthened literature review, and added/expanded content on social work contributions to animal shelter work. MO: researched and wrote preliminary literature review on concepts (compassion fatigue, burn out, etc.), and occupational stressors of animal shelter workers. All authors contributed to the article and approved the submitted version.

## Conflict of Interest

The authors declare that the research was conducted in the absence of any commercial or financial relationships that could be construed as a potential conflict of interest.

## Publisher's Note

All claims expressed in this article are solely those of the authors and do not necessarily represent those of their affiliated organizations, or those of the publisher, the editors and the reviewers. Any product that may be evaluated in this article, or claim that may be made by its manufacturer, is not guaranteed or endorsed by the publisher.
